# A green and environmentally benign route to synthesizing Z-scheme Bi_2_S_3_-TCN photocatalyst for efficient hydrogen production

**DOI:** 10.3389/fchem.2024.1340955

**Published:** 2024-02-02

**Authors:** Lang Yuan, Yihang Yin, Peng Xiang, Yugui Shao, Jie Gao, Jianan Liu, Huiyuan Meng, Li Li, Ying Xie, Xudong Xiao, Baojiang Jiang

**Affiliations:** ^1^ College of Modern Agriculture and Ecological Environment, Heilongjiang University, Harbin, China; ^2^ Heilongjiang Provincial Key Laboratory of Environmental Nanotechnology, School of Chemistry and Materials Science, Heilongjiang University, Harbin, China; ^3^ School of Safety Engineering, Heilongjiang University of Science and Technology, Harbin, Heilongjiang, China

**Keywords:** photocatalytic, heterojunction, TCN, Bi_2_S_3_, semiconductor

## Abstract

Designing and developing photocatalysts with excellent performance in order to achieve efficient hydrogen production is an important strategy for addressing future energy and environmental challenges. Traditional single-phase photocatalytic materials either have a large bandgap and low visible light response or experience rapid recombination of the photogenerated carriers with low quantum efficiency, seriously hindering their photocatalytic applications. To solve these issues, an important solution is to construct well-matched heterojunctions with highly efficient charge separation capabilities. To this end, an *in situ* sulfurization reaction was adopted after the deposition of Bi^3+^ supramolecular complex on a layered supramolecular precursor of tubular carbon nitride (TCN). X-ray diffraction (XRD) patterns confirmed that the as-prepared sample has a good crystalline structure without any other impurities, while high-resolution transmission electron microscopy (HR-TEM) revealed that the heterojunction possesses a 2D structure with a layer of nano-array on its surface. Combined Fourier-transform infrared (FT-IR) spectra and energy-dispersive X-ray spectroscopy (EDX) revealed the interfacial interactions. Owing to the formation of the Z-scheme heterojunction, the visible light adsorption and the separation efficiency of the photo-generated carriers are both obviously enhanced, leaving the high energy electrons and high oxidative holes to participate in the photocatalytic reactions. As a result, the photocatalytic hydrogen evolution rate of Bi_2_S_3_-TCN achieves 65.2 μmol g^-1^·h^-1^. This proposed green and environmentally benign route can also be applied to construct other sulfides with 2D TCN, providing some important information for the design and optimization of novel carbon-nitride-based semiconductors.

## 1 Introduction

The application of photocatalytic technology to produce hydrogen is an important means of addressing energy and environmental issues, with significant implications for achieving carbon neutrality and a benign carbon cycle. As a semiconductor, tubular carbon nitride (TCN) is widely used in photocatalysis due to its excellent thermodynamic stability, suitable band gap (∼2.7 eV), high surface area, and low cost (Cao et al., 2015; Cheng et al., 2021). However, as with traditional semiconductors, single-phase g-C_3_N_4_ exhibits low mobility of photogenerated electrons and holes under light radiation, leading to high recombination rates of charge carriers and low quantum efficiency (Thomas et al., 2008; Kumar et al., 2013). Additionally, TCN requires high light excitation energy, and it can only absorb and utilize ultraviolet and a narrow range of visible light less than 460 nm, resulting in unsatisfactory photocatalytic performance (Chen et al., 2014).

To address these issues, researchers have employed methods such as atomic-level doping (Zhang et al., 2011a; Pan et al., 2011), surface chemistry via molecular-level modification (Zhang et al., 2010; Chu et al., 2013), and construction of heterojunctions (Huang et al., 2022; Lei et al., 2022; Wang et al., 2022) to enhance the photocatalytic activity of TCN. Furthermore, the 2D layered structure and polymetric nature of TCN make it a very suitable host for constructing heterojunctions with various inorganic materials (Cheng et al., 2021). Therefore, choosing an appropriate semiconductor to form a desired composite with TCN has become an important strategy for developing inexpensive and efficient photocatalysts. Compared to metal oxides, metal sulfides usually have smaller band gaps, giving them superior visible light activity (Zhu et al., 2022). Sulfides such as CdS, ZnS, CuS, and Cd_x_Zn_1-x_S have been widely used as photocatalysts for hydrogen production due to their tunable band gaps and excellent visible light utilization (Zhang et al., 2011b; Li et al., 2013; Yuan et al., 2018). As a member of the sulfide family, Bi_2_S_3_ has a band gap of ∼1.3–1.7 eV (Chen et al., 2017; Liu et al., 2019) and also exhibits excellent visible light activity. When combined with other wide-bandgap semiconductors, Bi_2_S_3_ as a photosensitizer can effectively improve the photocatalytic performance of the composite (Chen et al., 2017). Moreover, non-toxic Bi_2_S_3_ has a layered structure, allowing it to easily form heterojunctions with 2D-structured TCN through surface chemical modification, which have been applied in the degradation of dyes and pollutants (Zhou et al., 2015; Chen et al., 2017; Yin et al., 2018; Hu et al., 2019; Liu et al., 2019; Gu et al., 2021; Okab and Alwared, 2023) and the reduction of CO_2_ (Guo et al., 2020), as well as efficiently killing multidrug-resistant bacteria (Li et al., 2019).

Although Bi_2_S_3_-TCN has some applications in the above fields, there are significant differences in the photocatalytic mechanisms reported, and its application in the field of photocatalytic hydrogen production is also rather limited (Li et al., 2023). To reveal the nature of interface interactions and the relevant photocatalytic mechanisms, this study employed a green and environmentally benign route to synthesize a Z-scheme Bi_2_S_3_-TCN heterojunction photocatalysts. The results indicate that the formation of a heterojunction interface promotes an obvious charge transfer between the two components, enhancing the separation efficiency of charge carriers. The interface interaction also helps further stabilize the crystal structure of Bi_2_S_3_, thus significantly expanding the visible light absorption capability of the heterojunction while inhibiting the photo-corrosion of sulfides. In addition, the Z-scheme band alignment ensures the reduction and oxidation capabilities of photogenerated electrons and holes respectively, synergistically leading to outstanding and stable photocatalytic hydrogen production.

## 2 Experimental section

### 2.1 Preparation of the samples

**Synthesis of the supramolecular precursors and TCN**. We dissolved 1.2 g of melamine in 75 mL of deionized water and stirred for 30 min; then 1.44 g of solid phosphorous acid was added with continuous stirring for another 60 min. The mixture was transferred into an autoclave lined with Teflon and kept at 180°C for 10 h. Finally, the product was filtered and washed ten times with deionized water. After drying at 60°C, the samples were placed in a magnetic boat and calcined at 450°C in a tube furnace for 2 h to obtain TCN.

**Synthesis of Bi**_**2**_ **S**_**3**_ **.** We uniformly dispersed 0.001 mol Bi(NO_3_)_3_·5H_2_O in 40 mL anhydrous ethanol and stirred for 2 h. After the hydrolysis of the precursor, the product was filtered and washed with ethanol three times and then dried at 60°C. Sublimed sulfur and the product were put into two magnetic boats, which were then transferred in the tube furnace and heated to 450°C with a heating rate of 2.5°C/min. After calcination for 2 h in an argon atmosphere, a Bi_2_S_3_ sample was obtained.

**Synthesis of Bi**_**2**_ **S**_**3**_ **-TCN.** We used 0.001 mol of Bi(NO_3_)_3_·5H_2_O as a Bi source to grow a Bi^3+^ supramolecular complex *in situ* on the surface of the layered supramolecular precursor of TCN (4 g) with anhydrous ethanol as solvent. After stirring for 2 h, the product was thrice filtered and washed with ethanol and dried at 60°C. Sublimed sulfur was then introduced as a sulfur source to vulcanize the precursor via vapor deposition calcination. Unlike the traditional hydrothermal preparation method, the growth of the Bi-based molecular complex on the 2D surface of TCN precursors not only guaranteed close interface contact between the two components but also reduced the reaction step, accompanied by an improved reaction yield and obviously reduced consumption of the organic chemical reagents. This synthetic method can effectively avoid the production of pollution and the waste of resources as far as possible and thus become a green and environmentally benign route.

### 2.2 Structural characterizations and photocatalytic tests

X-ray diffraction (XRD) patterns were obtained by a Bruker D8 diffractometer using Cu Kα radiation, while the Fourier transform infrared spectroscopy (DT-IR) data were recorded using the KBr pellets on a PerkinElmer Spectrum One spectrometer. The microstructures were identified by scanning electron microscopy (SEM, FEI Sirion 200) and transmission electron microscopy (FEI Talos F200S). Optical absorption and diffuse reflectance spectrometry (UV-DRS) were performed using a UV-Vis spectrophotometer (Lambda 950). The work function of samples was tested by Scanning Kelvin probe (SKP) (SKP5050 system, Scotland). The photoelectrochemical measurements were performed on a Princeton Versa STAT 2 electrochemical workstation with a standard three-electrode system (Ag/AgCl electrode reference electrode and Pt foil counter electrode). The relevant testing was conducted for the Mott–Schottky curves under natural light exposure with 0.5 M Na_2_SO_4_ as the electrolyte.

The photocatalytic hydrogen production experiments were performed in a photocatalytic hydrogen production system (Labsolar-6A, Beijing Perfect Light Technology Co., Ltd.). A cut-off filter (*λ* > 400 nm) was used to remove the UV light to produce visible light, and a 300 W Xe lamp was used as the light source. In a typical photocatalytic test, 50 mg of photocatalyst was dispersed in 100 mL of an aqueous solution containing 10 mL of lactic acid and 90 mL of water as the sacrificial reagent; the solution was stirred continuously during the test. Before visible light irradiation, the opening was sealed with a quartz cap with a silicone rubber gasket, and the test apparatus was evacuated for 30 min to remove O_2_ from the reaction system. Finally, the mixture was exposed under radiation and the gas product was analyzed using a gas chromatograph (Techcomp 7900, TCD, Ar carrier) to determine hydrogen production. The hydrogen production rate was calculated using the method of normalization of catalyst dosage and time.

## 3 Results and discussion

**FIGURE 1:**
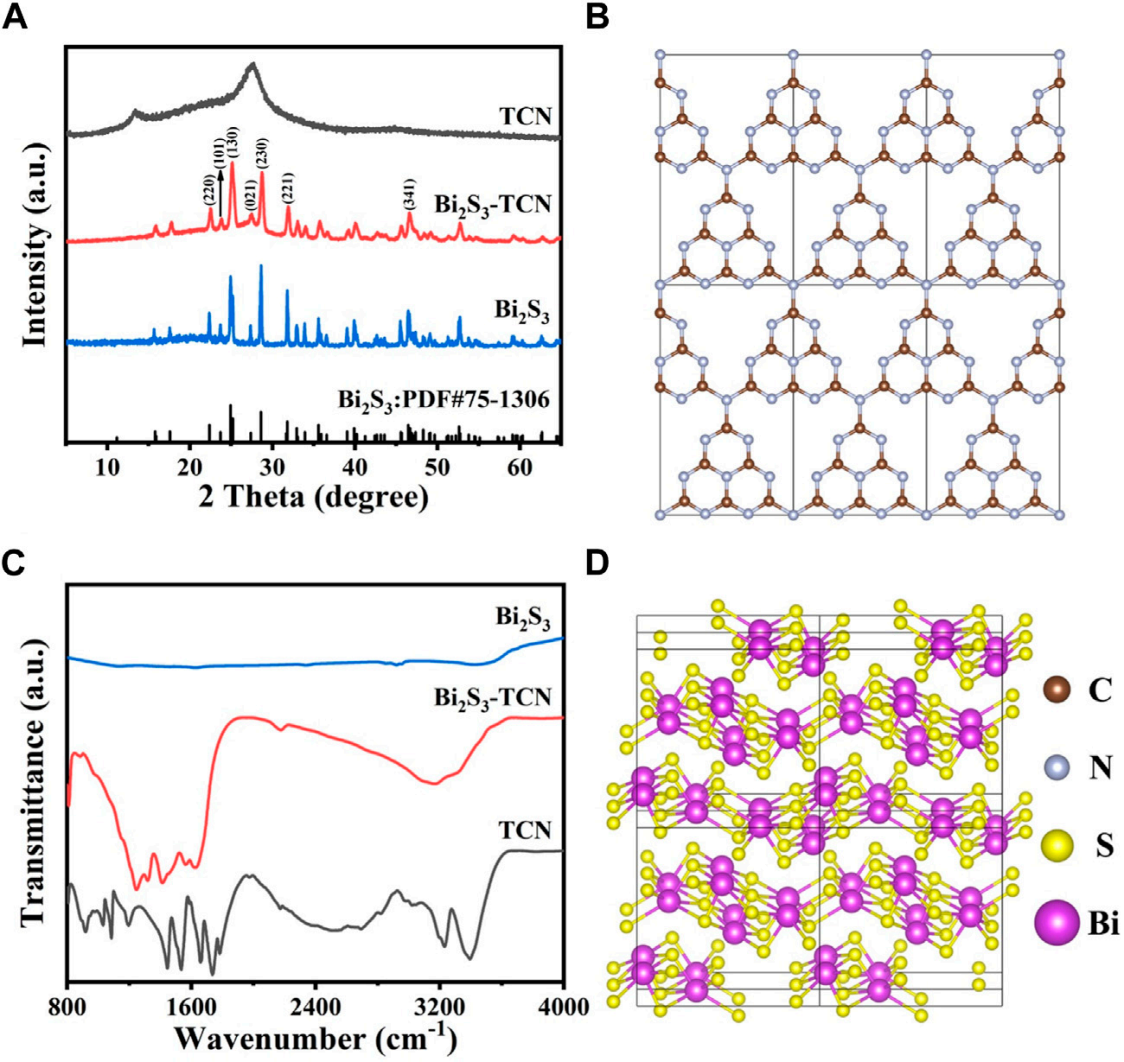
**(A)** XRD pattern for TCN, Bi_2_S_3_, and Bi_2_S_3_-TCN samples. **(B)** Crystal structures of TCN. **(C)** FT-IR spectrum for TCN, Bi_2_S_3_, and Bi_2_S_3_-TCN samples. **(D)** Crystal structures of Bi_2_S_3_.

To determine the crystalline structure, XRD patterns of different samples are presented in Figure 1A. The results indicate that TCN exhibits two diffraction peaks at approximately 13.0° and 28.0°, corresponding to the (100) and (002) crystal planes of graphitic carbon nitride (Li et al., 2022). The former primarily originates from the ordered repetitive arrangement of the in-plane tris-S-triazine structure of TCN (Figure 1B), while the latter is associated with the characteristic stacking of conjugated aromatic groups (Li et al., 2021; Sarkar et al., 2022). XRD peaks are sharp and clear for Bi_2_S_3_, indicating a good crystalline property. The diffraction peaks at 22.5°, 23.9°, 25.2°, 27.7°, 28.7°, 31.9°, and 46.7° correspond to (220), (101), (130), (021), (230), (221), and (341), and the crystal structure belongs to the orthorhombic system with a space group of P_nma_, which is consistent with the data in the standard card (PDF#75-1360, Figure 1A) (Lee et al., 2018; Sun et al., 2020). In addition, the diffraction peaks of both Bi_2_S_3_ and TCN are simultaneously observed in the Bi_2_S_3_-TCN sample, while the (002) diffraction peak of TCN and the (021) peak of Bi_2_S_3_ obviously overlap, resulting in a significant change of the intensities nearby. This demonstrates the coexistence of the two components in the composite and the successful construction of the Bi_2_S_3_-TCN heterojunction. The absence of XRD patterns for other impurities indicates the high purity of Bi_2_S_3_-TCN.

To reveal the impact of the introduction of Bi_2_S_3_ on the structure of TCN, Fourier-transform infrared (FT-IR) spectra of different samples are presented in Figure 1C. Corresponding to the building blocks in Figure 1B (Xiao et al., 2021), TCN exhibits a series of absorption bands (Yin et al., 2018): the absorption peak near 810 cm^-1^ corresponds to the stretching vibration of the triazine ring; the fingerprint signals within the range of 1,100–1,700 cm^-1^ correspond to the stretching vibrations of the C-N aromatic hetero-rings; the broad absorption band in the range of 3,000–3,600 cm^-1^ is mainly attributed to the stretching modes of primary and secondary amines, as well as the O–H stretching vibration of surface-adsorbed water and intermolecular hydrogen bonding interactions. Thanks to the layered structure of Bi_2_S_3_ (Figure 1D), its introduction may lead to the formation of heterojunctions with the TCN through interfacial interactions. This causes a slight shift of the above IR characteristic peaks toward lower wave numbers. However, there is no significant difference in the intensity of absorption peaks in the sample compared to the pristine TCN samples. The layered structure of TCN in the heterojunctions is therefore not destroyed by the introduction of Bi_2_S_3_, indicating good structural integrity for Bi_2_S_3_-TCN.

**FIGURE 2:**
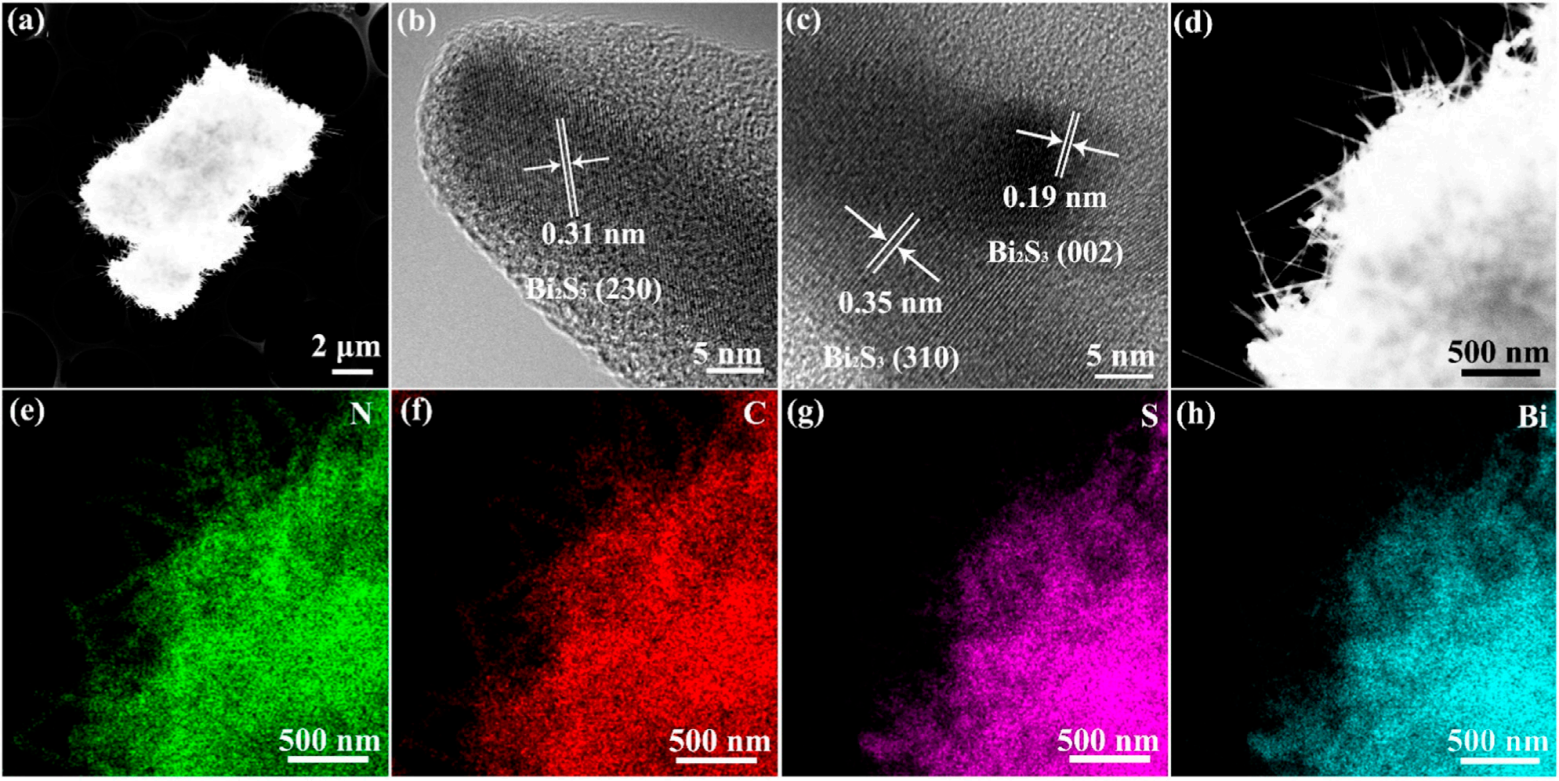
**(A–C)** High-resolution transmission electron microscopy (HR-TEM) images for Bi_2_S_3_-TCN heterojunctions. Energy dispersive X-ray spectroscopy (EDX) mapping for **(D)** Bi_2_S_3_-TCN, **(E)** N, **(F)** C, **(G)** S, and **(H)** Bi elements.

Figure 2A shows the high-resolution electron microscopy (HR-TEM) images and elemental distribution maps of the Bi_2_S_3_-TCN heterojunction. The results clearly show that the Bi_2_S_3_-TCN has a layered structure with special morphology. After assembly of the Bi^3+^ supramolecular complex on the TCN precursor surface, an *in situ* sulfurization process occurs and results in the formation of a layer of nano-array structures on the heterojunction surface. This effectively enhances the specific surface area and the number of active sites of the composite while simultaneously reducing the diffusion distance of photogenerated charge carriers. This is highly favorable for improving photocatalytic activity. In addition, the test results in Figures 2B,C further clarify that the crystal planes with the interplanar distances of 0.31 nm, 0.19 nm, and 0.35 nm belong to the Bi_2_S_3_ (230), (002), and (310) crystalline planes, respectively (Kumar et al., 2021). The EDX mapping further demonstrates a uniform distribution of the four elements (N, C, S, and Bi) throughout the whole selected region (Figures 2D–H). These features suggest that a well-contacted interface is formed between Bi_2_S_3_ and TCN in the heterojunction. Therefore, it can be anticipated that this heterojunction with a unique surface morphology and 2D structure will exhibit excellent photocatalytic performance.

**FIGURE 3:**
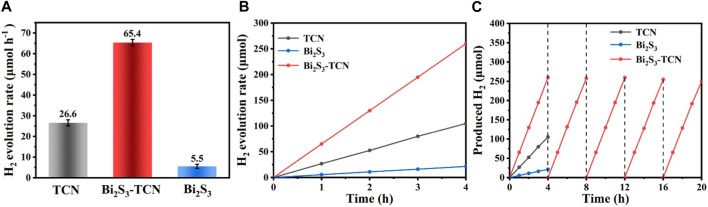
**(A)** H_2_ evolution rates for TCN, Bi_2_S_3_, and Bi_2_S_3_-TCN. **(B)** Time course of the photocatalytic H_2_ evolution of TCN, Bi_2_S_3_, and Bi_2_S_3_-TCN. **(C)** Recycling performance of TCN, Bi_2_S_3_, and Bi_2_S_3_-TCN.

The photocatalytic hydrogen production experiment was applied to evaluate the photocatalytic activity of the catalyst (Li et al., 2023). The hydrogen evolution rate of Bi_2_S_3_-TCN is 65.2 μmol h^-1^, which is 2.43 times that of TCN (26.8 μmol h^-1^) and 11.64 times that of Bi_2_S_3_ (5.6 μmol h^-1^). The mean values of the multiple experiments were Bi_2_S_3_-TCN (65.4 μmol h^-1^), TCN (26.6 μmol h^-1^), and Bi_2_S_3_ (5.5 μmol h^-1^), respectively (Figure 3A). As shown in Supplementary Table S1, the hydrogen evolution performance of Bi_2_S_3_-TCN was compared with existing heterojunction photocatalytic materials, demonstrating that Bi_2_S_3_-TCN had better photocatalytic hydrogen evolution activity. The significant increase in hydrogen production rate demonstrates that the formation of Bi_2_S_3_-TCN Z-scheme heterostructure provides a new charge transfer pathway with great advantages in photocatalytic reactions. At the same time, Bi_2_S_3_-TCN maintained a stable H_2_ yield during the 4-h test period (Figure 3B). The durability of Bi_2_S_3_-TCN was tested over five repeated cycles (Figure 3C). After 20 h of continuous testing, Bi_2_S_3_-TCN still maintained original photocatalytic activity, indicating that its heterojunction photocatalyst has excellent photostability. This indicates that the Bi_2_S_3_ and TCN heterojunction photocatalyst prepared can promote rapid separation and transfer of charge under light radiation while maintaining the stability of structure and morphology due to its tight heterogeneous structure.

**FIGURE 4:**
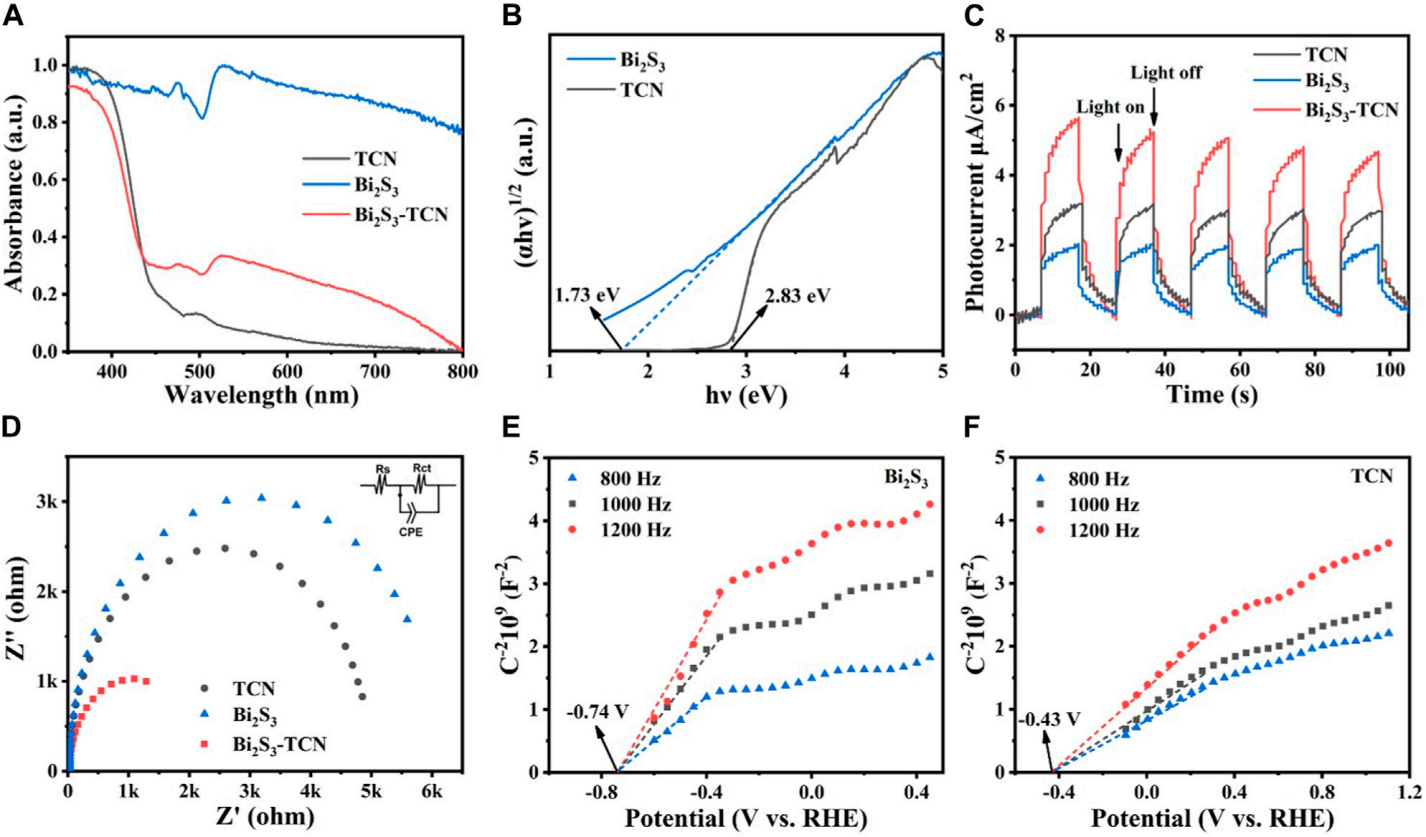
**(A)** Ultraviolet–visible diffuse reflectance spectroscopy (UV-Vis DRS), **(B)** optical band gaps, **(C)** transient photocurrent (TPC) plot, **(D)** electrochemical impedance spectroscopy (EIS), and **(E) (F)** Mott–Schottky plots of different samples.

To reveal the origin of the photocatalytic performance, UV-Vis DRS (Figure 4A) was used (Mua et al., 2021). It can be seen clearly that the absorption edge of TCN is close to 460 nm, while that of Bi_2_S_3_ extends significantly to 700 nm, indicating that Bi_2_S_3_ has much better visible light response. Compared with pristine TCN, the introduction of Bi_2_S_3_ leads to a red-shifted adsorption edge for the heterojunction. The interfacial interactions formed between TCN and Bi_2_S_3_ can modulate the positions of the valence band (VB) and conduction band (CB), obviously reducing the calculated band gap of Bi_2_S_3_-TCN (Figure 4B). Consequently, Bi_2_S_3_-TCN shows much stronger absorption of visible light, which is favorable for subsequent photocatalytic reactions. In addition to the above characteristics, the TPC plot in Figure 4C further identifies that the photocurrent of Bi_2_S_3_-TCN under visible light radiation is much higher than those of TCN and Bi_2_S_3_, and that even Bi_2_S_3_ possesses the smallest band gaps (Hao et al., 2020). This indicates that the intimate interface formed between the two components does facilitate the separation of the photocatalytic electrons and holes, leading to a much higher charge separation efficiency. Such a result can be further supported by the EIS data in Figure 4D (Xiao et al., 2022), in which the electric conductivity of Bi_2_S_3_-TCN with a minimal semi-circle is much better than Bi_2_S_3_ and TCN. Figures 4E,F show the Mott–Schottky plots for Bi_2_S_3_ and TCN, respectively. The positive slopes of the plots suggest that both materials are n-type semiconductors. The flat band potentials are found to be −0.74 and −0.43 V (vs RHE) for Bi_2_S_3_ and TCN, respectively, based on how the band alignment of the heterojunction is determined.

**FIGURE 5:**
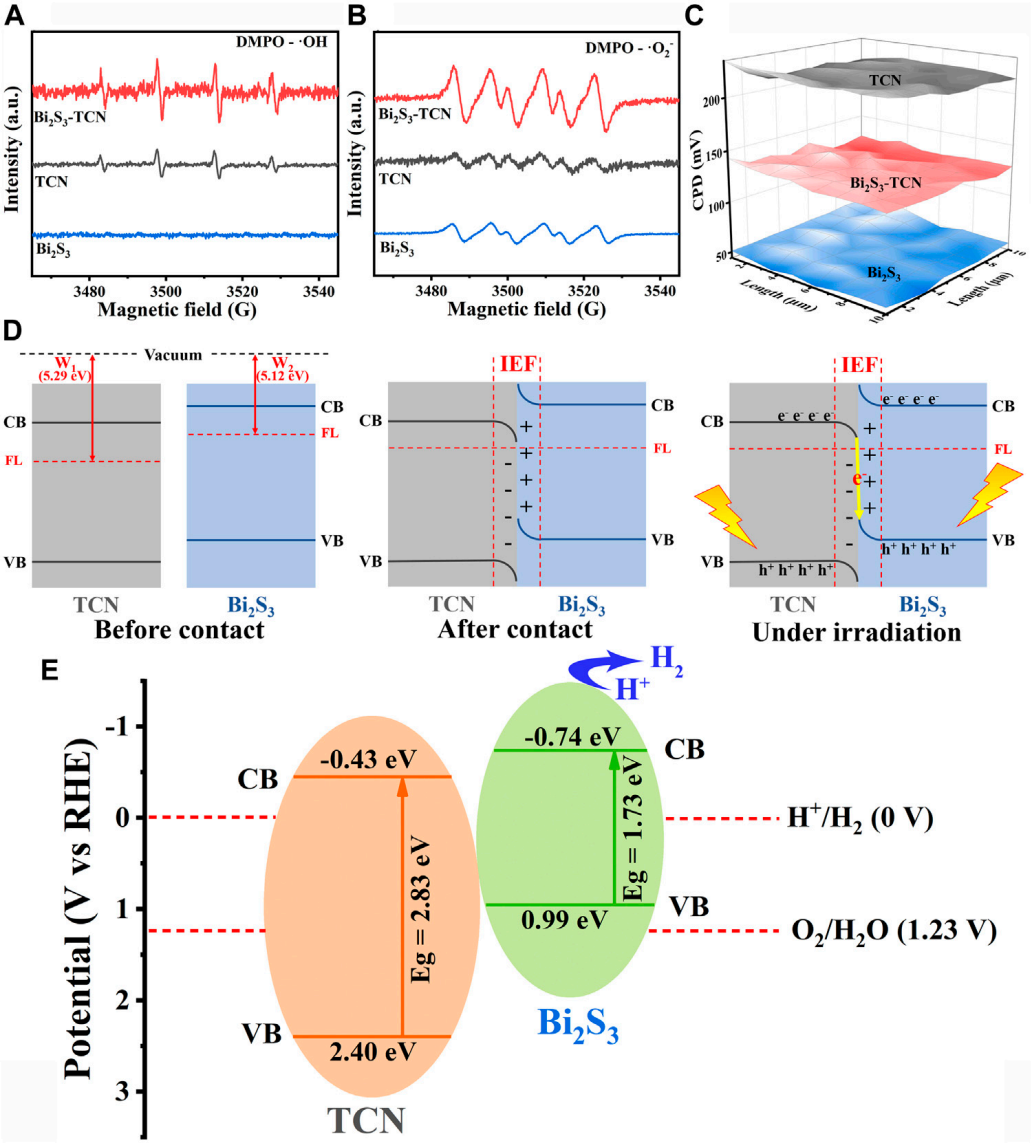
**(A, B)** ESR spectra of TCN, Bi_2_S_3_, and Bi_2_S_3_-TCN under visible-light illumination. **(C)** Relative WF maps of TCN, Bi_2_S_3_, and Bi_2_S_3_-TCN. **(D)** Energy band diagrams of TCN and Bi_2_S_3_ before and after contact, BIEF formation, and charge transfer processes in Z-scheme heterostructures. **(E)** Photocatalytic mechanism of Bi_2_S_3_-TCN heterojunctions.

X-ray photoelectron spectroscopy (XPS) was utilized to analyze the chemical makeup and bonding structure of the final product. The shift in peak value demonstrated the transfer of electronic structure between TCN and Bi_2_S_3_. The XPS analysis identified the presence of Bi and S in Bi_2_S_3_, and C and N in TCN and Bi_2_S_3_-TCN, respectively. Supplementary Figures S1, S2 demonstrate a noteworthy decline in the binding energy direction of Bi_2_S_3_-TCN’s C 1s and N 1s XPS peaks relative to TCN. Bi_2_S_3_-TCN’s 2p and Bi 4f spectra also show a positive shift in binding energy (Supplementary Figure S3). The XPS spectra results indicate that there is a close contact and charge transfer between Bi_2_S_3_ and TCN. We utilized the electron paramagnetic resonance (EPR) technique with 5,5-dimethyl-1-pyrroline N-oxide (DMPO) as the trapping agent of hydroxyl radical (•OH) (Figure 5A) to further confirm the charge transfer mechanism between Bi_2_S_3_ and TCN. Under visible light irradiation, four DMPO-•OH characteristic signal peaks with 1:2:2:1 intensity ratio of TCN and Bi_2_S_3_-TCN were observed, while no DMPO-•OH characteristic signal peaks appeared in Bi_2_S_3_. This indicates that the holes are concentrated in the VB of TCN and that only the VB level of TCN has sufficient oxidation capacity to reach •OH. Conversely, as shown in Figure 5B, the CB of both TCN and Bi_2_S_3_ is capable of reducing O_2_ to •O_2_^−^, leading to the detection of the •O_2_^−^ signal in all samples. Notably, the Bi_2_S_3_-TCN exhibits the most potent •O_2_^−^ signal, which can be ascribed to the recombination between TCN’s electrons and Bi_2_S_3_’s holes, resulting in more electrons accumulating in the CB of Bi_2_S_3_. It is possible that the electrons on the CB of TCN transfer to the VB of Bi_2_S_3_, creating a Z-scheme heterojunction photocatalytic system, with higher oxidation and reduction capacities located on TCN and Bi_2_S_3_, respectively. This suggests that photogenerated electrons and holes in Bi_2_S_3_-TCN may follow a Z-scheme electron transfer pattern instead of a type II electron transfer pattern (Supplementary Figure S4).

The work function (WF) of TCN, Bi_2_S_3_, and Bi_2_S_3_-TCN has been measured by Kelvin probe (Liu et al., 2022). The charge transfer direction in the composite photocatalyst can be well verified. As shown in Figure 5C, the contact potential difference (CPD) between TCN, Bi_2_S_3_, and Bi_2_S_3_-TCN and Au probes is 218 mV, 51 mV, and 130 mV, respectively, and the WF of TCN and Bi_2_S_3_ is calculated to be 5.29 and 5.12 eV, respectively (Figure 5D; Luo et al., 2023). When TCN and Bi_2_S_3_ are in contact, electrons are transferred from Bi_2_S_3_ to TCN through the contact interface until their Fermi levels reach equilibrium. This transfer method of electrons induces an embedded electric field between the positively charged Bi_2_S_3_ and negatively charged TCN at the interface. Consequently, carrier migration between Bi_2_S_3_ and TCN is accelerated. Within this particular embedded electric field, electrons accumulate at the TCN interface, while the electron density decreases at the Bi_2_S_3_ interface. This causes the TCN bands to bend downward and those on Bi_2_S_3_ to bend upward. Under illumination, the internal electric field and band bending facilitate the valence band transfer of conduction band electrons of tetracyanoquinodimethane to the boundary with Bi_2_S_3_, where they combine with the valence band hole of Bi_2_S_3_ to establish a Z-scheme electron transfer mechanism. This special charge transfer process accelerates the rate of carrier separation and transfer in the two components so that Bi_2_S_3_-TCN has a stronger REDOX capacity. As shown in Figure 5E, when both components in the heterojunction are excited under visible light radiation, the photocatalytic electrons in the CBM of TCN will diffuse and recombine with the electrons in the VBM of Bi_2_S_3_. With the formation of the S-scheme heterojunction, the high energy electrons in the CB of Bi_2_S_3_ and the high oxidative holes in the VB of TCN are retained (Liu et al., 2021). The participation of these highly active carriers in the following photocatalytic reactions are one important reason for the excellent photocatalytic hydrogen production of Bi_2_S_3_-TCN.

## 4 Conclusion

A green and environmentally benign route was adopted to successfully synthesize a Bi_2_S_3_-TCN heterojunction. The sample exhibits a 2D structure with a special surface morphology, and the intimate interface formed between Bi_2_S_3_ and TCN not only increases the photocatalytic charge separation efficiency but also extends the visible light adsorption of the sample, leading to a Z-scheme heterojunction with excellent photocatalytic performance. Bi_2_S_3_-TCN exhibits a hydrogen production rate of 65.2 μmol h^-1^, which is 11.64 and 2.43 times those of Bi_2_S_3_ and TCN.

## Data Availability

The original contributions presented in the study are included in the article/Supplementary Material; further inquiries can be directed to the corresponding authors.
