# Supplementary material for: A green and environmentally benign route to synthesizing Z-scheme Bi2S3-TCN photocatalyst for efficient hydrogen production
Source: Front Chem. 2024 Feb 2;12:1340955. doi: 10.3389/fchem.2024.1340955 (PMC10869476; doi:10.3389/fchem.2024.1340955)
Supplement: Supplementary file 1 [file DataSheet1.pdf]

Supporting Information

A Green and Environmentally Benign Route to Synthesize Z-scheme Bi_2S_3-TCN Photocatalyst for Efficient Hydrogen Production

Lang Yuan[1,2†], Yihang Yin[2†], Peng Xiang[2], Yugui Shao[2], Jie Gao[2], Jianan Liu[2], Huiyuan Meng[2,3] , Li Li [1,2]*, Ying Xie [2]*, Xudong Xiao [2]*, Baojiang Jiang [2]

[1] *College of Modern Agriculture and Ecological Environment, Heilongjiang University, Harbin 150080, P. R. China*

[2] *Heilongjiang Provincial Key Laboratory of Environmental Nanotechnology, School of Chemistry and Materials Science, Heilongjiang University, Harbin 150080, P. R. China.*

[3]*School of Safety Engineering, Heilongjiang University of Science and Technology, Harbin, Heilongjiang, P. R. China*

Co-cuthor[†]:
Lang Yuan, Yihang Yin

Correspondence*:
Corresponding Author
lili1993036@hlju.edu.cn, xxd@hlju.edu.cn, xieying@hlju.edu.cn

Keywords: Photocatalytic[1], Heterojunction[2], TCN[3], Bi_2S_3 [4], Semiconductor[5].

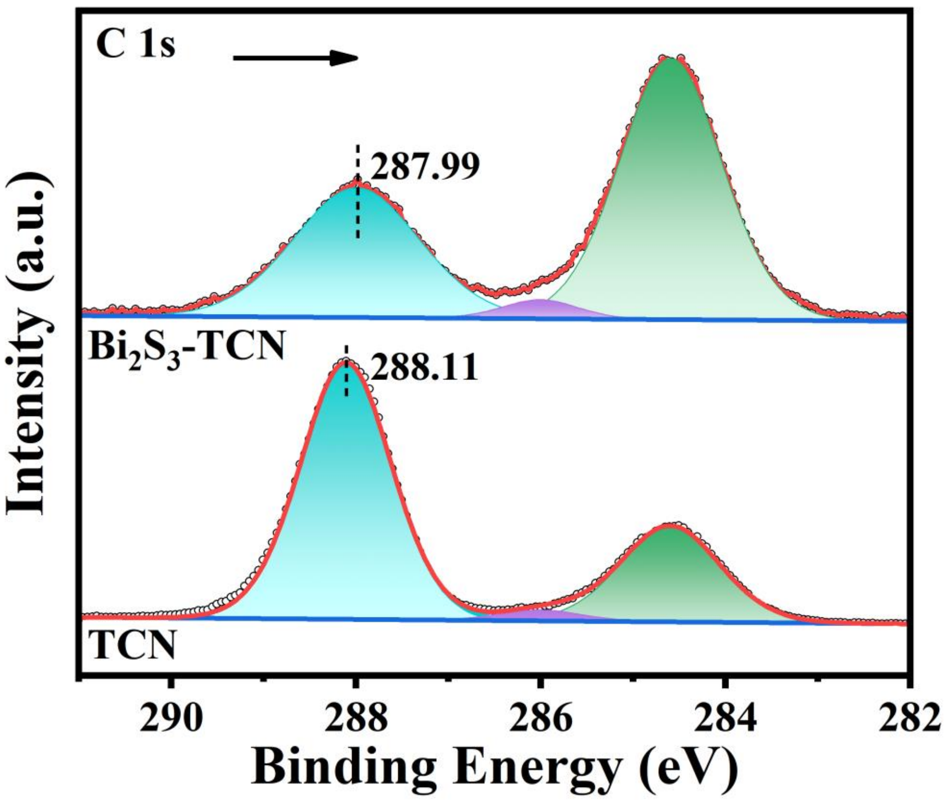

Figure S1. C 1s XPS of the TCN and Bi_2S_3-TCN.

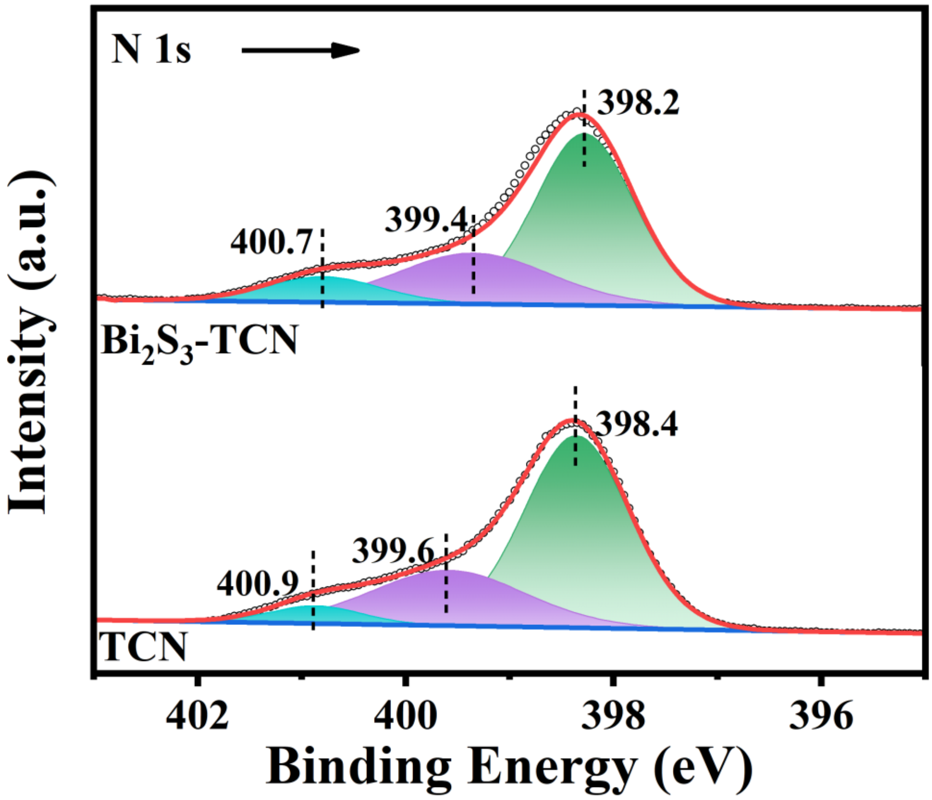

Figure S2. N 1s XPS of the TCN and Bi_2S_3-TCN.

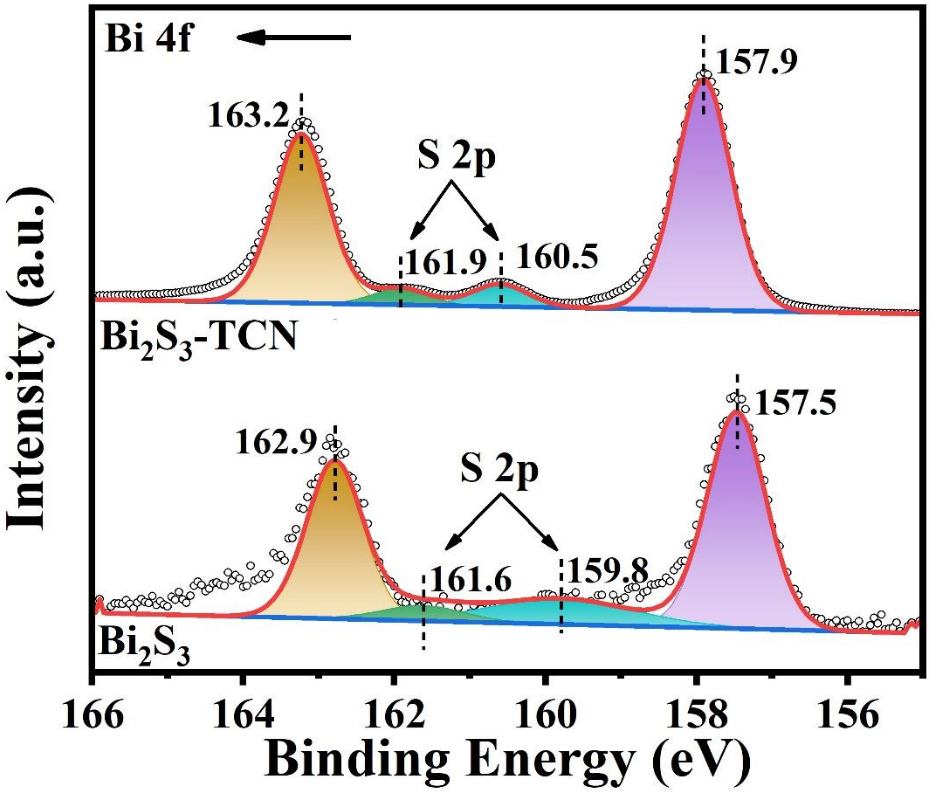

Figure S3. Bi 4f and S 2p XPS of the Bi_2S_3 and Bi_2S_3-TCN.

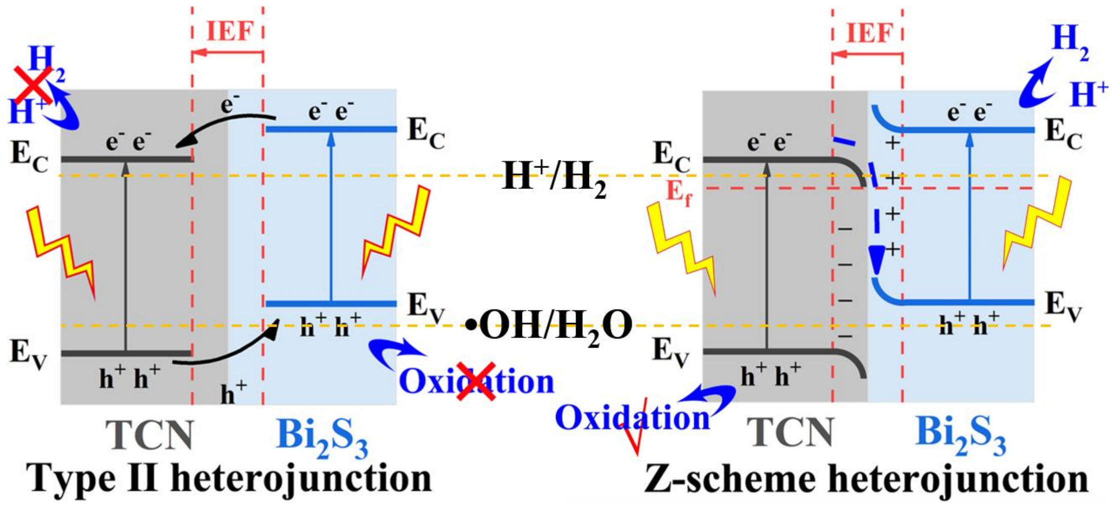

Figure S4. The energy level diagrams and charge transfer in Type II and Z-scheme Bi_2S_3-TCN heterostructure

Table S1. Comparison of hydrogen evolution performance between Bi_2S_3-TCN and heterojunction photocatalysts.

Materials	λ-value/Light source	H_2 production	AQE	Sacrificial agent	Ref
Bi_2S_3-TCN	λ > 400 nm, 300 W Xe-lamp	65.4 μmol h^{-1}		Lactic acid	This work
$Ni_{12}P_5$/g-C_3N_4	λ > 420 nm	632.8 μmol h^{-1} g^{-1}	–	TEOA	1
$AgIn_5S_8$/$ZnIn_2S_4$	λ > 420 nm	949.9 μmol h^{-1} g^{-1}	–	Methanol	2
CeO_2/$ZnIn_2S_4$	λ > 420 nm	847.42 μmol h^{-1} g^{-1}	–	Na_2S/Na_2SO_3	3
BP/g-C_3N_4	λ > 420 nm	271 μmol h^{-1} g^{-1}	–	Methanol	4
g-C_3N_4/$SnNb_2O_6$	λ > 420 nm	11.2 μmol h^{-1} g^{-1}	–	–	5
CoP/g-C_3N_4	λ < 420 nm, 300 W Xe-lamp	1.074 mmol h^{-1} g^{-1}	6.1%	TEOA	6
MoS_2/g-C_3N_4	λ > 420 nm	577 μmol h^{-1} g^{-1}	–	–	7
$SrTiO_3$/$SnNb_2O_6$	λ > 420 nm	5.72 μmol h^{-1}		methanol	8
CuS/Ti_3AlC_2	–	4.245 μmol h^{-1} g^{-1}		Na_2S/Na_2SO_3	9
Ni_2P/red P	λ > 420 nm	265.43 μmol h^{-1} g^{-1}		methanol	10
CoP/g-C_3N_4	λ > 420 nm	15.1 μmol h^{-1}	–	TEOA	11
FeP/g-C_3N_4	–	177.9 μmol h^{-1} g^{-1}	1.57%	TEOA	12
MoO_2–C/g-C_3N_4	λ > 400 nm, 300 W Xenon light	1071.03 μmol h^{-1} g^{-1}	–	TEOA	13
g-C_3N_4/g-C_3N_4	150 W metal halide lamp	241 μmol h^{-1} g^{-1}	–	TEOA	14
MoS_2/g-C_3N_4	λ > 420 nm	1286 μmol h^{-1} g^{-1}	1.2%	Na_2S/Na_2SO_3	15

REFERENCES

[1]. Dong, H., Hong, S., Zuo, Y., Zhang, X., Lu, Z., Han, J., Wang, L., Ni, L., Li, C., and Wang, Y. (2019). Fabrication of 2D/0D heterojunction based on the dual controls of micro/nanomorphology and structure towards high-efficiency photocatalytic H_2 production. Chem Cat Chem. 11(24),6263 – 6269. doi:10.1002/CCTC.201901618.

[2]. Guan, Z., Xu, Z., Li, Q., Wang, P., Li, G., and Yang, J. (2018). $AgIn_5S_8$ nanoparticles anchored on 2D layered $ZnIn_2S_4$ to form 0D/2D heterojunction for enhanced visible-light photocatalytic hydrogen evolution. Applied Catalysis B: Environmental. 227, 512-518. doi:10.1016/J.APCATB.2018.01.068.

[3]. Zhang, M., Yaom, J., Arif, M., Qiu, B., Yin, H., Liu, X., and Chen, S. (2020). 0D/2D $CeO_2/ZnIn_2S_4$ Z-scheme heterojunction for visible-light-driven photocatalytic H_2 evolution. Appl Surf Sci. 526, 145749. doi:10.1016/J. APSUSC.2020.145749.

[4]. Lei, W., Mi, Y., Feng, R., Liu, P., Hu, S., Yu, J., Liu, X., Rodriguez, J. A., Wang, J., Zheng, L., Tang, K., Zhu, S., Liu, G., and Liu, M. (2018). Hybrid 0D – 2D black phosphorus quantum dots – graphitic carbon nitride nanosheets for efficient hydrogen evolution. Nano Energy. 50, 552 – 561. doi:10.1016/J.NANOEN.2018.06.001.

[5]. Luo, B., Hong, Y., Li, D., Fang, Z., Jian, Y., ang Shi, W. (2018). Fabrication of 0D/2D carbon nitride quantum dots/$SnNb_2O_6$ ultrathin nanosheets with enhanced photocatalytic hydrogen production. ACS Sustainable Chem Eng. 6, 14332 – 14339. doi:10.1021/ACSSUSCHEMENG.8B03006

[6]. Luo, B., Song, R., Geng, J., Liu, X., Jing, D., Wang, M., and Cheng, C. (2019). Towards the prominent cocatalytic effect of ultra-small CoP particles anchored on g-C_3N_4 nanosheets for visible light driven photocatalytic H_2 production. Applied Catalysis B: Environmental. 256, 117819. doi:10.1016/J.APCATB.2019.117819.

[7]. Liu, Y., Zhang, H., Ke, J., Zhang, J., Tian, W., Xu, X., Duan, X., Sun, H., O, Tade .M., and 1QWang, S. (2018). 0D (MoS_2)/2D (g-C_3N_4) heterojunctions in Z-

scheme for enhanced photocatalytic and electrochemical hydrogen evolution. Applied Catalysis B: Environmental. 228, 6474. doi:10.1016/J.APCATB.2018.01.067.

[8]. Jin, Y., Jiang, D., Li, D., Xiao, P., Ma, X., and Chen, M. (2017). $SrTiO_3$ nanoparticle/$SnNb_2O_6$

nanosheet 0D/2D heterojunctions with enhanced interfacial charge separation and photocatalytic hydrogen evolution activity. ACS Sustainable Chem Eng. 5, 9749 – 57. doi:10.1021/ACSSUSCHEMENG.7B01548.

[9]. Xie, Y., Rahman, MM., Kareem, S., Dong, H., Qiao, F., Xiong, W., Liu, X., Li, N., and Zhao, X. (2020) Facile synthesis of CuS/MXene nanocomposites for efficient photocatalytic hydrogen generation. CrystEngComm. 22, 2060 – 2066. doi:10.1039/ D0CE00104J.

[10]. Liang, Z., Dong, X., Han, Y., and Geng, J. (2019). In-situ growth of 0D/2D Ni_2P quantum dots/red phosphorus nanosheets with p-n heterojunction for efficient photocatalytic H_2 evolution under visible light. Appl Surf Sci. 484, 293 – 299. doi:10.1016/J.APSUSC.2019.04.006.

[11]. Han, C., Zhang, T., Cai, Q., Ma, C., Tong, Z., and Liu, Z. (2019). 0D CoP cocatalyst/2D g-C_3N_4 nanosheets: an efficient photocatalyst for promoting photocatalytic hydrogen evolution. J Am Ceram Soc. 102,5484 – 93. doi:10.1111/ JACE.16443.

[12]. Zeng, D., Zhou, T., Ong, W. J., Wu, M., Duan, X., Xu, W., Chen, Y., Zhu, Y., and Peng, D. Sub-5 nm ultra-fine FeP nanodots as efficient Co-catalysts modified porous g-C_3N_4 for precious-metal-free photocatalytic hydrogen evolution under visible light. ACS Appl Mater Interfaces. 11,5651 – 60. doi:10.1021/ ACSAMI.8B20958/.

[13]. Chen, Z., Xia, K., She, X., Mo, Z., Zhao, S., Yi, J., Xu, Y., Chen, H., Xu, H., and Li, H. (2018). 1D metallic MoO_2-C as co-catalyst on 2D g-C_3N_4 semiconductor to promote photocatlaytic hydrogen production. Appl Surf Sci. 447, 732 – 739.

doi:10.1016/J. APSUSC.2018.03.226.

[14]. Mahzoon, S., Nowee, S.M., and Haghighi, M. （2018） Synergetic combination of 1D-2D g-C_3N_4 heterojunction nanophotocatalyst for hydrogen production via water splitting under visible light irradiation. Renew Energy. 127, 433 – 443. doi: 10.1016/J.RENENE.2018.04.076.

[15]. Yuan, Y., Wang, P., Li, Z., Wu, Y., Bai ,W., Su, Y., Guan, J., Wu, S., Zhong, J., Yu, Z., and Zou, Z. (2019) The role of bandgap and interface in enhancing photocatalytic H_2 generation activity of 2D-2D black phosphorus/MoS_2 photocatalyst. 242, 18. doi:10.1016/J.APCATB.2018.09.100.
